# ^18^F-FP-PEG_2_-β-Glu-RGD_2_: A Symmetric Integrin α_v_β_3_-Targeting Radiotracer for Tumor PET Imaging

**DOI:** 10.1371/journal.pone.0138675

**Published:** 2015-09-23

**Authors:** Kongzhen Hu, Xiaolan Tang, Ganghua Tang, Shaobo Yao, Baoguo Yao, Hongliang Wang, Dahong Nie, Xiang Liang, Caihua Tang, Shanzhen He

**Affiliations:** 1 Department of Nuclear Medicine, the First Affiliated Hospital, Sun Yat-Sen University, Guangzhou, 510080, China; 2 College of Materials and Energy, Southern China Agricultural University, Guangzhou, 510642, China; National Center for Scientific Research Demokritos, GREECE

## Abstract

Radiolabeled cyclic arginine-glycine-aspartic (RGD) peptides can be used for noninvasive determination of integrin α_v_β_3_ expression in tumors. In this study, we performed radiosynthesis and biological evaluation of a new ^18^F-labeled RGD homodimeric peptide with one 8-amino-3,6-dioxaoctanoic acid (PEG_2_) linker on the glutamate β-amino group (^18^F-FP-PEG_2_-β-Glu-RGD_2_) as a symmetric PET tracer for tumor imaging. Biodistribution studies showed that radioactivity of ^18^F-FP-PEG_2_-β-Glu-RGD_2_ was rapidly cleared from blood by predominately renal excretion. MicroPET-CT imaging with ^18^F-FP-PEG_2_-β-Glu-RGD_2_ revealed high tumor contrast and low background in A549 human lung adenocarcinoma-bearing mouse models, PC-3 prostate cancer-bearing mouse models, and orthotopic transplanted C6 brain glioma models. ^18^F-FP-PEG_2_-β-Glu-RGD_2_ exhibited good stability *in vitro* and *in vivo*. The results suggest that this tracer is a potential PET tracer for tumor imaging.

## Introduction

**Fig 1:**
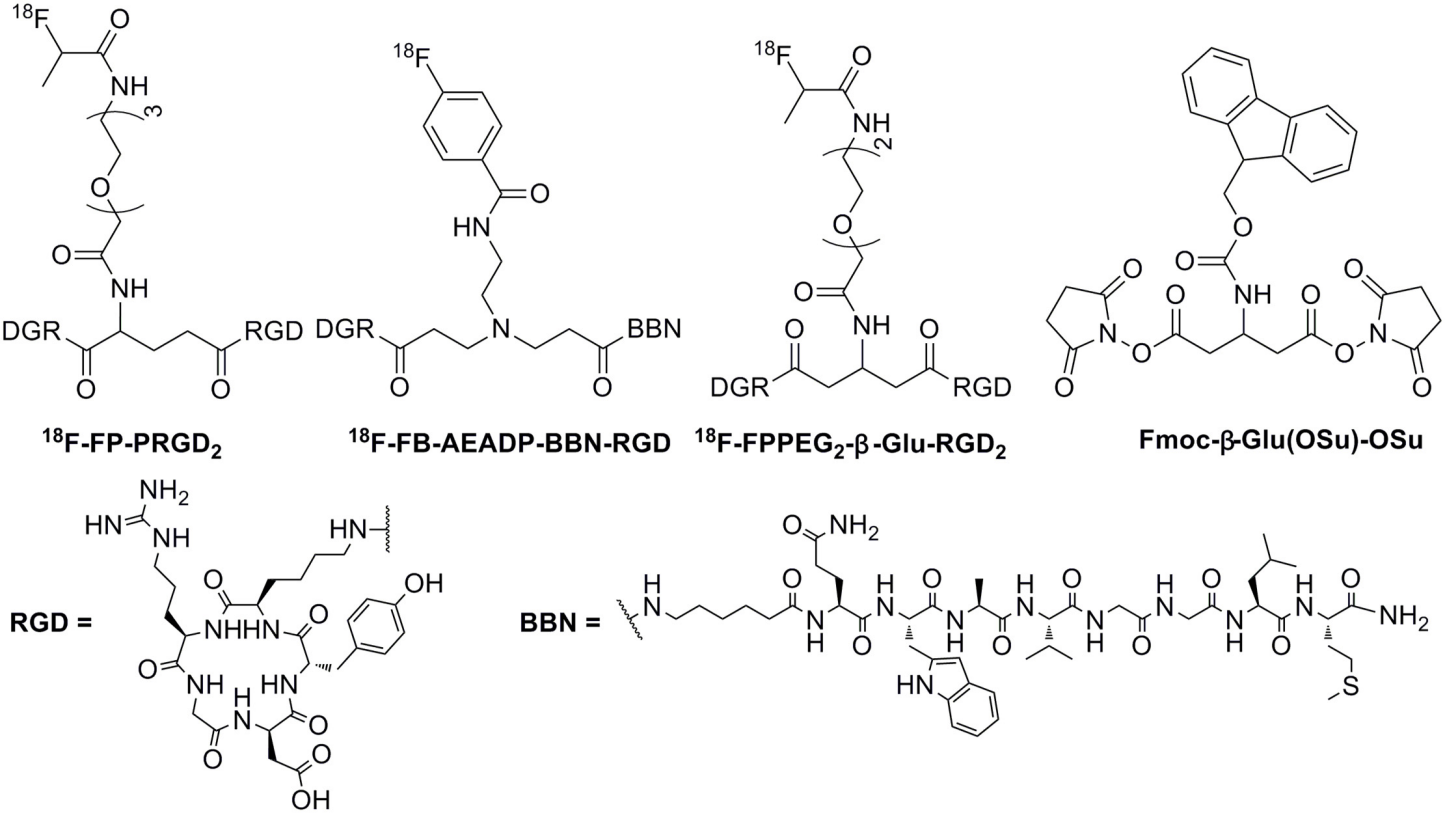
Chemical structures of ^18^F-PP-PRGD_2_, ^18^F-FB-AEADP-BBN-RGD, ^18^F-FP-PEG_2_-β-Glu-RGD_2_, and Fmoc-β-Glu(OSu)-OSu.

Angiogenesis is the process of formation of new vessels in avascular tissue. It plays a key role in a variety of processes such as rheumatoid arthritis [1], psoriasis [2], cardiovascular diseases [3], diabetic retinopathy [4], and tumor growth, as well as tumor metastasis [5]. The angiogenic process depends on vascular endothelial cells (ECs) migration and invasion [6]. Integrin α_v_β_3_ involves in the migration of ECs during formation of new blood vessels and is highly expressed only in activated ECs of tumor neovasculature, not in normal cells or quiescent ECs [7]. Monomeric cyclic arginine-glycine-aspartic (RGD) peptides and analogs showed high affinity and selectivity for the integrin α_v_β_3_ [8]. Thus, several radiolabeled monomeric RGD peptides and analogs have been developed for monitoring and quantifying integrin α_v_β_3_ expression noninvasively in tumors with positron emission tomography (PET) [9–16]. For example, ^18^F-galacto-RGD [11] and ^18^F-AH111585 [10] have been developed in clinical studies for PET imaging of integrin α_v_β_3_. To further improve the binding affinity of peptide-based probes, multimeric RGD peptides have been reported and shown that the dimeric RGD peptides as imaging agents, as exemplified by ^18^F-FP-PRGD_2_ (Fig 1) for clinical PET imaging of integrin α_v_β_3_ [9], are better than monomeric, tetrameric and octameric RGD peptides [12–14]. Thus, many radiolabled dimeric RGD peptides with asymmetric α-glutamate (Glu) linker group have been prepared and evaluated as integrin α_v_β_3_-targeted radiotracers [14–16]. Symmetric linkers provided a convenient method to synthesize dual-receptor-targeting tracers, such as ^18^F-FB-AEADP-BBN-RGD [AEADP = 3,3’-(2-aminoethy- lazanediyl)-dipropanoic acid] (Fig 1) [17]. However, as far as we know, the radiolabeled symmetric cyclic RGD dimeric peptide as integrin α_v_β_3_-targeting radiotracers have not been reported yet.

We previously reported a symmetric β-glutamate linker Fmoc-β-Glu(OSu)-OSu for preparation of ^18^F-FP-PEG_3_-β-Glu-RGD_2_ (Fig 1), showing Fmoc-β-Glu(OSu)-OSu as a good symmetric linker for coupling homodimers and heterodimers [18–20]. In this study, we designed a new symmetric RGD homodimer peptide PEG_2_-β-Glu-RGD_2_, which was labeled with ^18^F (97% β^+^ decay; maximum β^+^ energy = 0.64 MeV, t_1/2_ = 109.8 min) by conjugation with 4-nitrophenyl 2-^18^F-fluoropropionate (^18^F-NFP). The symmetrical ^18^F-labeled PEG_2_-β-Glu-RGD_2_ (^18^F-FP-PEG_2_-β-Glu-RGD_2_) (Fig 1) was then used for noninvasive imaging of integrin α_v_β_3_ expression in tumor-bearing animals to evaluate its usefulness.

## Materials and Methods

### Chemicals and Equipment

All chemicals purchased were of analytical grade and used without further purification unless otherwise indicated. RGD was purchased from APeptide Co., Ltd (Shanghai, China). Mass spectrometry (MS) was obtained on a Quattro/LC mass spectrometer by electro-spray ionization. SEP-PAK light QMA and Oasis HLB cartridges were obtained from Waters Corporation (Milford, MA, USA). SEP-PAK light QMA cartridges were preconditioned with 5 mL NaHCO_3_ aqueous (8.4%) and 10 mL water before use. Oasis HLB cartridges were preconditioned with 10 mL ethanol and water before use. Analytical HPLC was performed using an Agilent 1200 Series HPLC system equipped with a Easeatech AQ-C18 (4.6 × 150 mm, 5 μm; Welch Materials, Inc) at the flow rate of 1 mL/min. The gradient program started from 98% solvent A (0.1% trifluoroacetic acid in water): 2% solvent B (0.1% trifluoroacetic acid in MeCN) ramped to 90% solvent A: 10% solvent B at 8 min, and ramped to 20% solvent A: 80% solvent B at 20 min. The elution profile was detected with an ultraviolet detector (Agilent interface 35900E, Agilent Technologies, USA) at 254 nm and a B-FC-3200 high energy PMT Detector (Bioscan. Washington DC, USA). Radioactivity was measured by a calibrated ion chamber (Capintec CRC-15R) or a gamma counter (γ-counter) (GC-1200, USTC Chuangxin Co. Ltd. Zonkia Branch, China).

### Synthesis of FP-PEG_2_-β-Glu-RGD_2_

**Fig 2:**
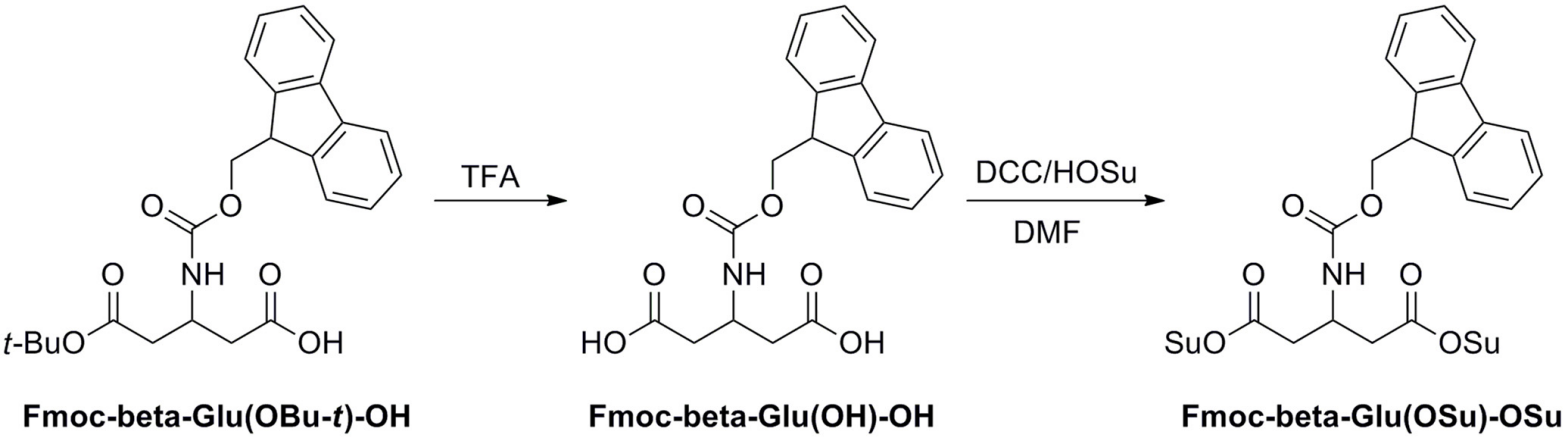
Synthetic route of Fmoc-β-Glu(OSu)-OSu.

The Fmoc-protected β-glutamic acid activated ester linker Fmoc-β-Glu(OSu)-OSu was prepared according to Fig 2 as previously reported [19]. Fmoc-beta-Glu(OtBu)-OH (100 mg, 0.235 mmol) was hydrolyzed with trifluoroacetic acid (TFA) and isolated with the HPLC system to give Fmoc-beta-Glu(OH)-OH. After the collected fractions were combined and lyophilized to give a powder, to a solution of Fmoc-beta-Glu(OH)-OH in 2 mL of *N*, *N*-dimethylforamide (DMF) were added *N*-hydroxysuccinimide (HOSu) (0.126 mg, 1.10 mmol) and dicyclohexylcarbodiimide (DCC) (0.226 mg, 1.10 mmol). The resulting mixture was stirred at room temperature for 10 h. The dicyclohexylurea by-product was filtered off. The filtrate was evaporated to dryness under vacuum to give a crude product, which was then taken up in 3 mL of methylene chloride. The insoluble solid was filtered off. The filtrate was concentrated to about 1 mL. The solution was added dropwise into 30 mL of ether. The desired product was precipitated as white solid, which was dried in vacuo. The yield was 60% (79.4 mg, 0.141 mmol). MS (ESI, m/z): 564.2 ([M+H]^+^). ^1^H-NMR (CDCl_3_) δ (ppm): 7.22–7.82 (m, 8H, 9-fluorenyl aromatic hydrogen), 5.34 (s, 1H, NH), 4.86 (m, 1H, CH), 4.68 (d, 2H, COOCH_2_), 4.40 (m, 1H, COOCH_2_CH), 2.64 (m, 8H, succinimide group), 2.42 (d, 4H, CH_2_CO).

**Fig 3:**
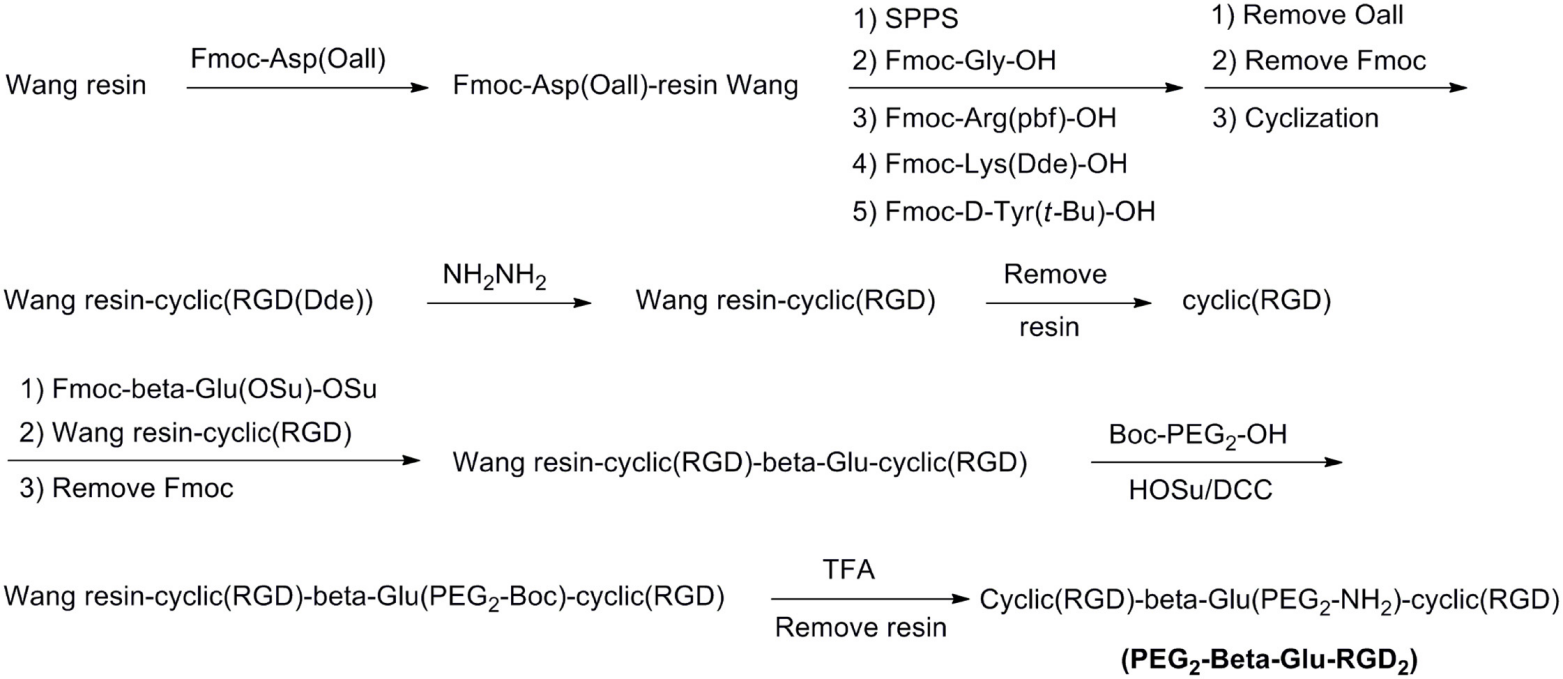
The route of PEG_2_-β-Glu-RGD_2_ via solid-phase peptide synthesis (SPPS).

The PEG_2_-β-Glu-RGD_2_ homodimer peptide was synthesized by APeptide Co., Ltd (Shanghai, China) according to solid-phase peptide synthesis (SPPS) strategy as described in Fig 3. MS (ESI, m/z): 1495.8 ([M+H]^+^) [19].

Synthesis of FP-PEG_2_-β-Glu-RGD_2_ was performed by reaction of PEG_2_-β-Glu-RGD_2_ with 4-nitrophenyl 2-fluoropropionate (NFP). In brief, to a solution of anhydrous NFP (1 mg, 4.7 μmol) in anhydrous DMSO (200 μL) was added PEG_2_-β-Glu-RGD_2_ (1 mg, 0.67 μmol in 600 μL of DMSO) and DIPEA (40 μL). The reaction mixture was stirred for 2 h at 50°C. After quenching with 0.5 mL 5% acetic acid, the final product was purified by preparative HPLC and lyophilized to afford FP-PEG_2_-β-Glu-RGD_2_ as a white powder. MS (ESI, m/z): 1570.4 ([M+H]^+^).

### Radiosynthesis of ^18^F-FP-PEG_2_-β-Glu-RGD_2_

**Fig 4:**
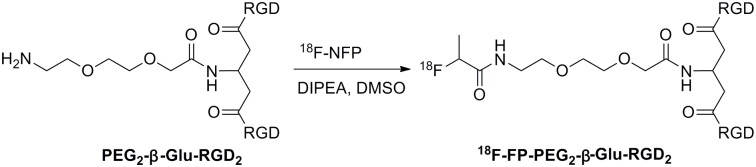
Radiosynthesis of ^18^F-FP-PEG_2_-β-Glu-RGD_2_.

No-carrier-added ^18^F-fluoride was obtained through the nuclear reaction ^18^O (p, n)^18^F by irradiation of a more than 95% ^18^O-enriched water target with a 10-MeV proton beam on the PET trace cyclotron (IBA Technologies, Belgium). ^18^F-NFP was synthesized according to the reported procedure [20,21]. To the dried ^18^F-NFP residue was added a solution of PEG_2_-β-Glu-RGD_2_ (100 μg) in 20 μL of DIPEA and 0.2 mL of anhydrous DMSO (Fig 4). The reaction mixture was heated to 40°C for 5 min, and then was quenched with 0.5% acetic acid (1 mL) and water (10 mL). The mixture solution was passed through an Oasis HLB cartridge and the cartridge was washed with water (10 mL). The desired product was eluted from the cartridge with 2 mL of ethanol. The solvent was removed by a stream of nitrogen at 50°C. The ^18^F-labeled peptide was formulated in normal saline and passed through a 0.22 μm Millipore filter into a sterile vial for experiments.

### Cells and Animals

This study was carried out in strict accordance with the recommendations in the Guide for the Care and Use of Laboratory Animals of the Ministry of Science and Technology of the People's Republic of China. The experimental protocols were approved by the Committee on the Ethics of Animal Experiments of the First Affiliated Hospital, Sun Yat-Sen University (Permit Number: [2012]001). All surgeries were performed under chloral hydrate anesthesia, and all efforts were made to minimize suffering, to reduce the number of animals used, and to use alternatives to *in vivo* techniques, if available.

Cell lines and mice were obtained from the Laboratory Animal Center of Sun Yat-Sen University. The cells were cultivated in RPMI 1640 medium with a physiologic glucose concentration (1.0 g/L) containing 5% fetal calf serum at 37°C in a humidified atmosphere of 5% CO_2_ and 95% air. In the study, 20 normal mice, 20 nude mice and 5 rats were used. Among them, sixteen normal mice were used for biodistribution analysis, four normal mice were used for *in vivo* metabolism, and twenty nude mice and five rats were used for making tumor-bearing models. Mice or rats were housed 5 animals per cage under standard laboratory conditions at 25°C and 50% humidity. Every day mice and rats were observed for signs of ill health and no animal death was found. Eight PC-3 tumor-bearing models were generated by subcutaneous injection of 5 × 10^6^ tumor cells into the right shoulder of male athymic nude mice. Twelve A549 human lung adenocarcinoma-bearing models were generated by subcutaneous injection of 2 × 10^6^ tumor cells into the left shoulder of male athymic nude mice. Five orthotopic transplanted C6 brain glioma models were made by injection of 2 × 10^6^ tumor cells into the brain of rat. MicroPET-CT studies were performed on the mice 1–4 weeks after inoculation when the tumor diameter reached 0.6–1.0 cm (3–4 weeks after inoculation for PC-3 models and C6 brain glioma models, and 1–2 weeks for A549 models).

### Biodistribution Studies

For single-isotope (^18^F) biodistribution studies, sixteen normal Kunming mice or eight A549 lung adenocarcinoma-bearing nude mice were injected with 1.48–2.96 MBq (40–80 μCi) of ^18^F-FP-PEG_2_-β-Glu-RGD_2_ in 100–200 μL of saline through the tail vein. The mice were kept anesthetized with 5% chloral hydrate solution after tracer administration. Radioactivity in the syringe before and after administration was measured in a calibrated ion chamber. The animals were sacrificed by cervical dislocation at various times after injection, blood was obtained through the eyeball vein, the organs of interest (blood, brain, heart, lung, liver, kidney, pancreas, spleen, stomach, and intestine) were rapidly dissected and weighed, and ^18^F radioactivity was counted with a γ-counter. All measurements were background-subtracted and decay-corrected to the time of injection, then averaged together. Data were expressed as a percentage of the injected dose per gram of tissue (%ID/g) (n = 4 per group).

### *In Vitro* Stability and *in Vivo* Metabolism

For the *in vitro* experiment, a sample of ^18^F-FP-PEG_2_-β-Glu-RGD_2_ (1.48 MBq, 10 μL) dissolved in normal saline was added to 200 μL of mouse serum and incubated at 37°C. An aliquot of the serum sample was passed through a 0.22 μm Millipore filter and injected into a radio-HPLC column to analyze the stability of ^18^F-FP-PEG_2_-β-Glu-RGD_2_ in mouse serum within 2 h. The experiment was performed using 3 separate samples. The metabolic stability of ^18^F-FP-PEG_2_-β-Glu-RGD_2_ was evaluated in normal Kunming mice (n = 3). Each mouse was injected with ^18^F-FP-PEG_2_-β-Glu-RGD_2_ at dose of 3.7–14.8 MBq (100–400 μCi) in saline via a tail vein. After 30 min post-injection, the urine was carefully collected and analyzed by radio-HPLC.

### MicroPET-CT Imaging

PET imaging of tumor-bearing mice was carried out using the Inveon small animal PET/computed tomography (CT) scanner (Siemens). 3.7 MBq (100 μCi) of ^18^F-FP-PEG_2_-β-Glu-RGD_2_ was injected intravenously in conscious animals via the tail vein. A few minutes later the mice were anesthetized with 5% chloral hydrate solution (6 mL/kg). Ten-minute static PET images were acquired at four time points (30, 60, 90, and 120 min) postinjection. The images were reconstructed by two-dimensional ordered-subset expectation maximum (OSEM). For the integrin receptor-blocking experiment, RGD (4 mg/kg) was injected with 3.7 MBq of ^18^F-FP-PEG_2_-β-Glu-RGD_2_ into PC-3 tumor-bearing mice (n = 4). At 1 h after injection, the 10-min static microPET scans were acquired. For each microPET scan, regions of interest (ROIs) were drawn over the tumor, normal tissue, and major organs on decay-corrected whole-body coronal images using Inevon Research Workplace 4.1 software. The maximum radioactivity concentration (accumulation) within a tumor or an organ was obtained from mean pixel values within the multiple ROI volume, which was converted to MBq/mL/min by using a conversion factor. Assuming a tissue density of 1 g/mL, the ROIs were converted to MBq/g/min and then divided by the administered activity to obtain an imaging ROI-derived %ID/g. The mice were sacrificed at the end of the study for PET imaging.

### Statistical Analysis

Data were expressed as mean ±SD. Statistical analysis was performed with SPSS software, version 13.0 (SPSS Inc.), for Windows (Microsoft). A P value of less than 0.05 was considered to indicate statistical significance.

## Results

### Chemistry and Radiochemistry

The symmetric RGD homodimer peptide PEG_2_-β-Glu-RGD_2_ was synthesized by using Fmoc/t-Bu-protected solid-phase peptide synthesis (Fig 3). Resin-cyclic(RGD) was coupled with symmetric Fmoc-β-Glu(OSu)-OSu linker (Fig 2) and further reacted with cyclic(RGD) peptide to afford resin-cyclic(RGD)-β-Glu(Fmoc)- cyclic(RGD). After removal of the Fmoc-group, Boc-PEG_2_-OH was attached to the free primary amine of the β-Glu group in resin-cyclic(RGD)-β-Glu-cyclic(RGD). The resin-peptide was released from the solid support and protecting groups were removed by TFA treatment to afford homodimer peptide PEG_2_-β-Glu-RGD_2_. PEG_2_-β-Glu-RGD_2_ was isolated by semi-preparative HPLC and lyophilized. The purity and identity of the final product was confirmed by HPLC and ESI-MS spectrometry [19].

^18^F-Radiolabeling of PEG_2_-β-Glu-RGD_2_ homodimer with ^18^F-NFP was performed in DMSO for 5 min (Fig 4). The desired product was purified by an Oasis HLB cartridge, which greatly reduced the total synthesis time. The decay-corrected radiochemical yield was 60 ± 10% (n = 8) from ^18^F-NFP with high radiochemical purity (> 95%). The total radiochemical yield of ^18^F-FP-PEG_2_-β-Glu-RGD_2_ was 18 ± 3% (n = 10, decay-uncorrected) based on ^18^F-fluoride within 110 min and the specific radioactivity of ^18^F-FP-PEG_2_-β-Glu-RGD_2_ was more than 114.0 GBq/μmol. The radiochemical purity of ^18^F-FP-PEG_2_-β-Glu-RGD_2_ was over 95%.

### *In vivo* Biodistribution

The biodistribution data of ^18^F-FP-PEG_2_-β-Glu-RGD_2_ in normal Kunming mice are summarized in Table 1. The radioactivity had fast clearance from blood. ^18^F-FP-PEG_2_-β-Glu-RGD_2_ cleared predominantly through the renal pathway as evidenced by high kidneys uptake and rapid washout, with 14.0 ± 3.7%ID/g, 4.66 ± 1.23%ID/g, 2.23 ± 0.20%ID/g, and 0.73 ± 0.07%ID/g at 5, 30, 60, and 120 min postinjection, respectively. The liver showed moderate uptake of radioactivity and a relatively slow washout rate. Other tissues including brain, spleen, bone, stomach, and muscle, showed relatively low uptake and rapid clearance of radioactivity. ^18^F-FP-PEG_2_-β-Glu-RGD_2_ showed the similar biodistribution and pharmacokinetics characteristics to the previous reported RGD_2_ radiotracers [14,16].

**Table 1:** Biodistribution of ^18^F-FP-PEG_2_-β-Glu-RGD_2_ in Normal Mice^a^.

Organ	5min	30min	60min	120min
Blood	4.66 ± 0.57	1.65 ± 0.52	1.25 ± 0.18	0.48 ± 0.15
Brain	1.71 ± 0.28	0.86 ± 0.23	0.85 ± 0.25	0.30 ± 0.08
Heart	2.40 ± 0.45	1.07 ± 0.39	0.66 ± 0.13	0.34 ± 0.09
Lung	2.80 ± 0.24	1.32 ± 0.58	1.13 ± 0.45	0.39 ± 0.15
Liver	3.55 ± 1.12	2.01 ± 0.67	1.79 ± 0.33	0.81 ± 0.16
Pancreas	2.20 ± 0.25	0.97 ± 0.39	0.54 ± 0.28	0.23 ± 0.06
Kidney	14.0 ± 3.7	4.66 ± 1.23	2.23 ± 0.20	0.73 ± 0.07
Spleen	1.88 ± 0.68	0.98 ± 0.19	0.64 ± 0.07	0.42 ± 0.15
Intestine	2.03 ± 0.95	1.18 ± 0.17	1.17 ± 0.33	0.91 ± 0.31
Muscle	1.65 ± 0.75	0.85 ± 0.25	0.71 ± 0.24	0.38 ± 0.18
Stomach	1.37 ± 0.41	0.92 ± 0.13	0.86 ± 0.23	0.71 ± 0.05
Bone	1.68 ± 0.28	0.96 ± 0.37	0.73 ± 0.07	0.40 ± 0.06

^a^Means ± SD (n = 4). Data are average % ID/g.

Biodistribution of ^18^F-FP-PEG_2_-β-Glu-RGD_2_ in A549 lung adenocarcinomamice- bearing mice is shown in Table 2. ^18^F-FP-PEG_2_-β-Glu-RGD_2_ had avid uptake (3.38 and 2.68% ID/g) in A549 tumor at 30 and 60 min postinjection, respectively. The A549 tumor-to-muscle ratio (target-to-background ratio) decreased slightly from 3.60 at 30 min to 3.12 at 60 min postinjection. Biodistribution studies also demonstrated high kidneys uptake and rapid washout. Bone uptake was relatively low, suggesting no *in vivo* defluorination of the tracer.

**Table 2:** Biodistribution of _18_F-FP-PEG_2_-β-Glu-RGD_2_ in Nude Mice Bearing A549 Xenografts^a^.

Organ	30min	60min
Blood	2.12 ± 0.55	1.59 ± 0.57
Brain	1.62 ± 0.08	1.41 ± 0.14
Heart	1.99 ± 0.19	1.47 ± 0.03
Lung	2.04 ± 0.12	1.65 ± 0.10
Liver	3.14 ± 0.04	2.35 ± 0.26
Pancreas	1.48 ± 0.14	1.15 ± 0.08
Kidney	4.63 ± 1.04	2.66 ± 0.25
Spleen	2.55 ± 0.15	1.72 ± 0.13
Intestine	2.49 ± 0.34	1.64 ± 0.13
Muscle	0.94 ± 0.18	0.86 ± 0.06
Stomach	1.61 ± 0.04	1.22 ± 0.21
Bone	1.17 ± 0.21	0.88 ± 0.10
Tumor	3.38 ± 0.23	2.68 ± 0.25
Tumor to Muscle ratio	3.60	3.12

^a^Means ± SD (n = 4). Data are average % ID/g.

### Stability and Metabolism

Radio-HPLC analysis demonstrated that ^18^F-FP-PEG_2_-β-Glu-RGD_2_ in serum was proven to be stable. ^18^F-FP-PEG_2_-β-Glu-RGD_2_ was kept more than 95% intact in mouse serum at 37°C for 2 h. The stability *in vivo* and fate of ^18^F-FP-PEG_2_-β-Glu-RGD_2_ over 30 min was analyzed in urine. There were almost no metabolites of ^18^F-FP-PEG_2_-β-Glu-RGD_2_ detectable in urine, suggesting that ^18^F-FP-PEG_2_-β-Glu-RGD_2_ is metabolically stable during its excretion via the renal route.

### MicroPET-CT Imaging Study

**Fig 5:**
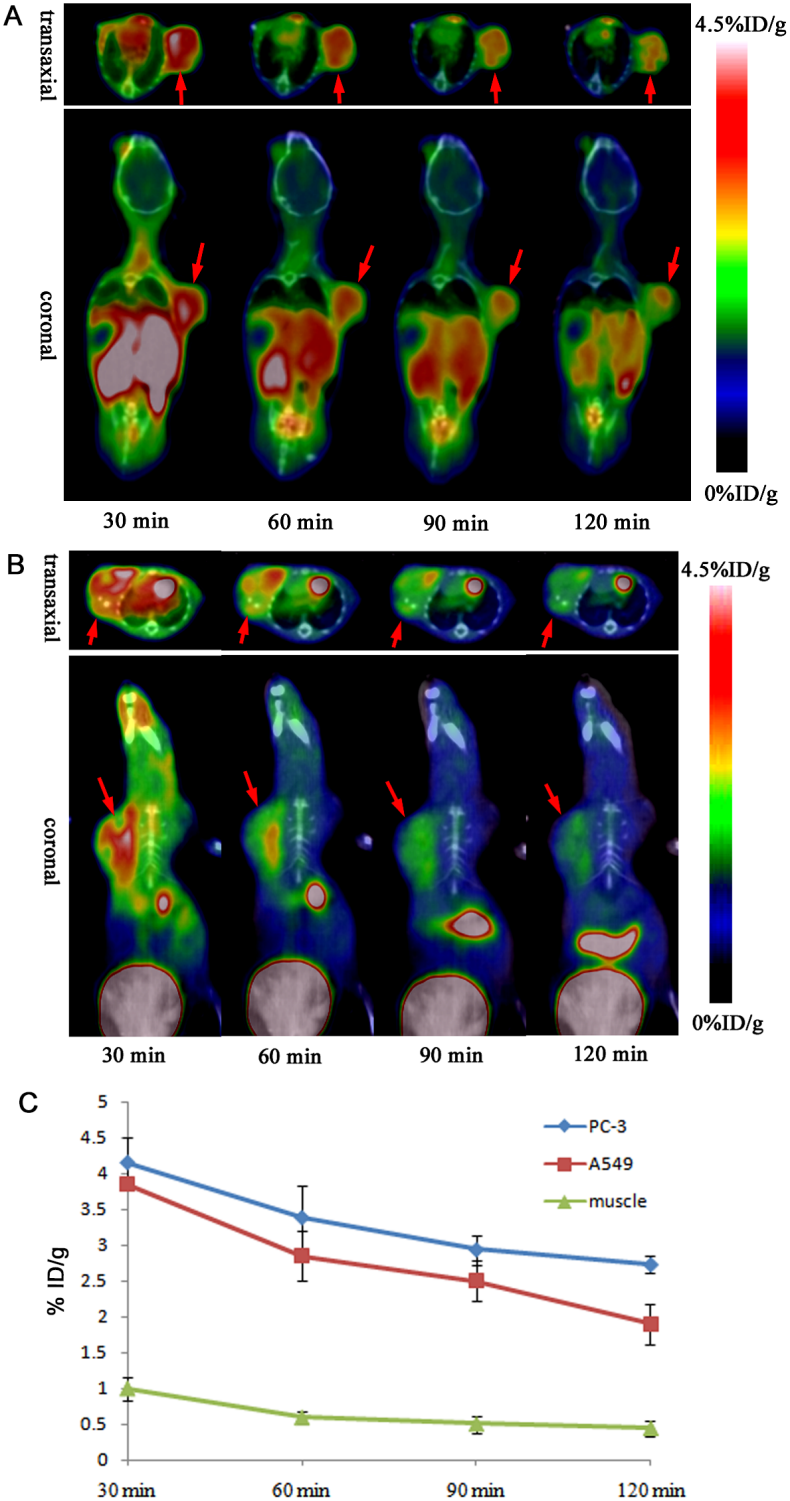
Small animal PET imaging and quantification. (A) MicroPET-CT imaging of PC-3 prostate tumor-bearing mice at 30, 60, 90, and 120 min after injection of ^18^F-FP-PEG_2_-β-Glu-RGD_2_. (The red arrows indicated PC-3 prostate tumor). (B) MicroPET-CT imaging of A549 human lung adenocarcinoma-bearing mice at 30, 60, 90, and 120 min after injection of ^18^F-FP-PEG_2_-β-Glu-RGD_2_. (The red arrows indicated A549 lung adenocarcinoma). (C) Time-activity curves of PC-3 prostate tumor, A549 lung adenocarcinoma and muscle after intravenous injection of ^18^F-FP-PEG_2_-β-Glu-RGD_2_.

MicroPET-CT imaging was carried out with ^18^F-FP-PEG_2_-β-Glu-RGD_2_ in PC-3 prostate cancer-bearing mouse models (Fig 5A) and A549 human lung adenocarcinoma-bearing mouse models (Fig 5B). The tumors were clearly visible with high contrast to the contralateral background at all time points measured from 30 to 120 min and had the highest uptake at 30 min postinjection of ^18^F-FP-PEG_2_-β-Glu-RGD_2_. High renal uptake at early time points was found and bladder accumulation was also observed, suggesting that the tracer is mainly excreted via the renal-bladder route. Radioactivity accumulations in tumors and muscles were quantified by measuring the ROIs encompassing the entire organ on the coronal PET images (Fig 5C). The results demonstrated that ^18^F-FP-PEG_2_-β-Glu-RGD_2_ had significantly higher uptake in tumors than that in muscles and also had high tumor/muscle uptake ratios. In addition, the tumor uptake of ^18^F-FP-PEG_2_-β-Glu-RGD_2_ in PC-3 prostate tumor-bearing mice was higher than that in A549 human lung adenocarcinoma-bearing mice (3.38 ± 0.44% ID/g vs. 2.85 ± 0.35%ID/g, n = 4, P < 0.05) at 60 min postinjection (Fig 5C), possibly due to higher level expression of integrin α_v_β_3_ in PC-3 prostate cancer than that in A549 human lung adenocarcinoma.

**Fig 6:**
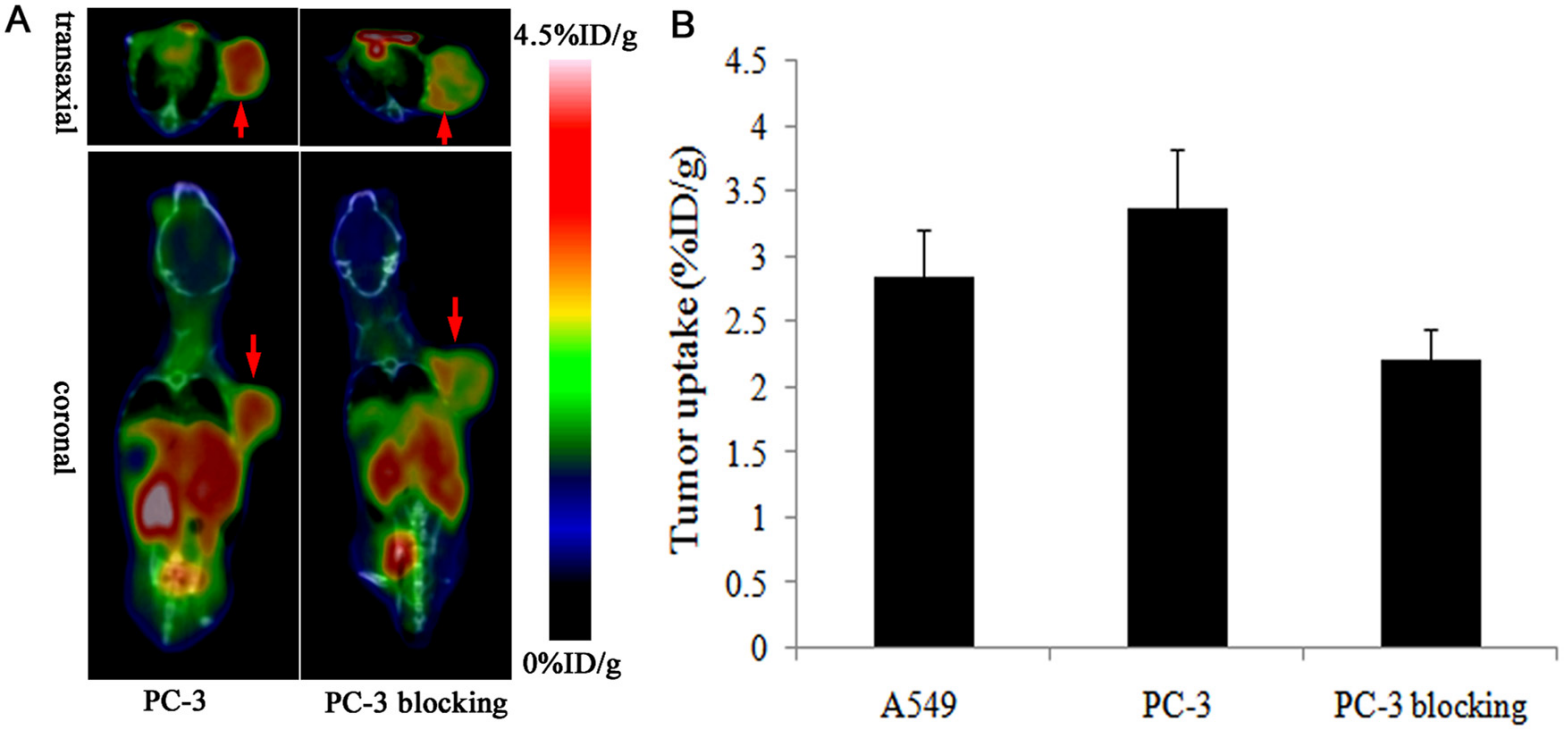
Tumor uptake of ^18^F-FP-PEG_2_-β-Glu-RGD_2_ was inhibited by co-injection of an excess dose of RGD. (A) MicroPET-CT images of PC-3 prostate tumor-bearing mice without/with a blocking dose of RGD (4 mg/kg body weight) at 60 min after injection of ^18^F-FP-PEG_2_-β-Glu-RGD_2_. (The red arrows indicated PC-3 prostate tumor). (B) The tumor uptake of ^18^F-FP-PEG_2_-β-Glu-RGD_2_ in PC-3 prostate tumor and PC-3 prostate tumor treated with cold RGD blocking at 60 min after injection (mean ±SD, n = 4 per group).

The integrin α_v_β_3_ binding specificity of ^18^F-FP-PEG_2_-β-Glu-RGD_2_ in PC-3 prostate tumor was confirmed by blocking studies (Fig 6). As shown in Fig 6A and 6B, the tumor uptake of ^18^F-FP-PEG_2_-β-Glu-RGD_2_ at 1 h postinjection was significantly inhibited by co-injection of an excess dose of RGD (3.38 ± 0.44%ID/g for no blocking vs. 1.82 ± 0.23%ID/g for blocking, n = 4 per group, P < 0.05), which is obvious evidence that there is high expression of integrin α_v_β_3_ receptor in the tumor.

**Fig 7:**
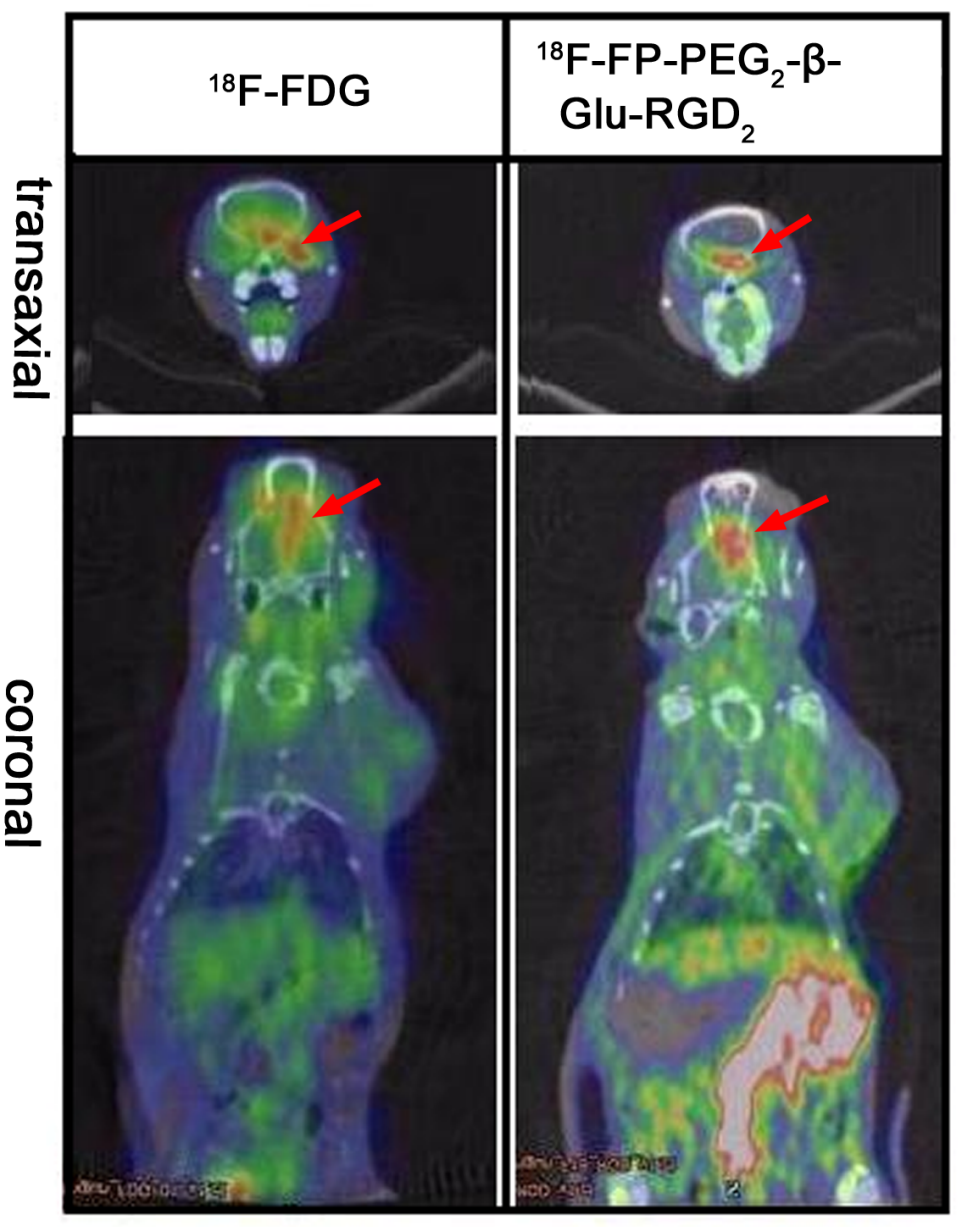
MicroPET-CT fused images of orthotopic transplanted C6 brain glioma model rats at 60 min post-injection of ^18^F-FDG and ^18^F-FP-PEG_2_-β-Glu-RGD_2_. (The red arrows indicate the tumor).

PET images of orthotopic-transplanted brain glioma rat models are shown in Fig 7. A low uptake of ^18^F-FP-PEG_2_-β-Glu-RGD_2_ was detected in normal brain tissue, but a high uptake in brain glioma tissue was observed. At 60 min post-injection of ^18^F-FP-PEG_2_-β-Glu-RGD_2_, uptake ratio of glioma tissue to white matter and glioma tissue to gray matter was 2.63 ± 0.53%ID/g and 1.61 ± 0.32%ID/g respectively, which were a little higher than those of ^18^F-FDG (2.24 ± 0.42%ID/g and 1.45 ± 0.21%ID/g, n = 5, P> 0.05, respectively). The results demonstrate that ^18^F-FP-PEG_2_-β-Glu-RGD_2_ is a useful PET tracer for brain cancer imaging.

## Discussion

Based on our previous works [18–20], we used a symmetric β-glutamate linker Fmoc-β-Glu(OSu)-OSu to prepare the symmetric ^18^F-FP-PEG_2_-β-Glu-RGD_2_ peptide as an integrin α_v_β_3_-targeting PET radiotracer. β-Glutamic acid was a substrate for glutamine synthetase in biosynthesis [22,23]. The symmetric PEG_n_-β-Glu linker was used to improve *in vivo* pharmacokinetic properties of drugs [24]. In our work, the Fmoc-β-Glu(OSu)-OSu linker was comprised of one Fmoc-protecting amino group and two activated carboxylic acid ester groups. The protruding free PEG-amino group in PEG_2_-β-Glu-RGD_2_ encountered less steric hindrance for ^18^F-labeling with ^18^F-NFP than that in PEG-α-Glu-RGD_2_ [9,16]. Compared with the asymmetric α-glutamic acid activated ester group (α-glutamate linker), the symmetric β-glutamic acid activated ester group (β-glutamate linker) could be used to prepare symmetric homodimer peptides by conjugating with the free amino groups of the peptides via forming amide bonds. Also, the symmetric β-glutamate linker was easy to prepare only one heterodimer product with definite pure compound structure due to the symmetric nature of the linker. In comparison with the existing tracer ^18^F-FP-PRGD_2_, having the overall uncorrected radiochemical yield of 10–15% (n = 5) starting from ^18^F^−^ and the measured specific activity of 114 ± 72 GBq/μmol within a total synthesis time of around 3 h [25], ^18^F-FP-PEG_2_-β-Glu-RGD_2_ gave a little high radiochemical yield (18 ± 3%, decay-uncorrected) based on ^18^F-fluoride and specific radioactivity of more than 114.0 GBq/μmol within a short total synthesis time of 110 min. In addition, the symmetric Fmoc-β-Glu(OSu)-OSu linker could exhibit definite non-toxicity and could be easily prepared without tedious synthesis, compared with the symmetric AEADP linker, which was recently used for coupling peptide heterodimer [17]. Therefore, the Fmoc-β-Glu(OSu)-OSu linker provides a general method for fast assembly of various peptide homodimers and heterodimers.

^18^F-FP-PRGD_2_ is a potential asymmetric RGD homodimer PET tracer, recently approved by the FDA (IND 104150) for a first-in-human test [9,14]. In the RGD dimer the close two cyclic RGD motifs may lead to the enhanced integrin α_v_β_3_ binding rate or the reduced dissociation rate of the cyclic RGD peptide from the integrin α_v_β_3_ [8]. Similar structure to asymmetric ^18^F-FP-PRGD_2_ [14,15], ^18^F-FP-PEG_2_-β-Glu-RGD_2_ with symmetric β-glutamate linker instead of asymmetric α-glutamate linker of ^18^F-FP-PRGD_2_ does not affect the binding affinity of RGD_2_ homodimer to integrin α_v_β_3_. Especially, the two RGD motifs of the symmetric RGD dimer peptide (^18^F-FP-PEG_2_-β-Glu-RGD_2_) are equally binding to the integrin α_v_β_3_ receptor and may significantly increase “local RGD concentration” of the receptor-binding site. Thus, symmetric ^18^F-FP-PEG_2_-β-Glu-RGD_2_ should possess better binding affinity to integrin α_v_β_3_ and higher accumulation in tumors than ^18^F-FP-PRGD_2_; but this needs to be further investigated. The *in vivo* blocking experiment demonstrated that a coinjection of excess amounts of RGD significantly inhibited uptake of ^18^F-FP-PEG_2_-β-Glu-RGD_2_ in the PC-3 tumor, confirming the integrin α_v_β_3_-binding specificity *in vivo*. In addition, comparison of the PET imaging results of ^18^F-FP-PRGD_2_ and ^18^F-FP-PEG_2_-β-Glu-RGD_2_ revealed that they had comparable tumor uptake and non-specific tissue uptake at the different tumor-bearing models [14].

The biodistribution results showed high uptake of ^18^F-FP-PEG_2_-β-Glu-RGD_2_ in the kidneys and in tumors (PC-3 prostate tumor and A549 human lung adenocarcinoma), low radioactivity accumulation in other organs, and rapid clearance from the body, which were similar to the observed biodistribution results of ^18^F-FP-PRGD_2_ in U87MG tumor [14]. The *in vivo* biodistribution results of ^18^F-FP-PEG_2_-β-Glu-RGD_2_ were also correlated well with the microPET data of model mice. Also, ^18^F-FP-PEG_2_-β-Glu-RGD_2_ exhibited excellent stability *in vitro* and *in vivo*. No defluorination of ^18^F-FP-PEG_2_-β-Glu-RGD_2_ was observed, as no visible bone uptake was found in mice biodistribution and in microPET imaging of model mice. These observations were consistent with other RGD homodimer radiotracers reported previously [14–16]. In addition, PET images of orthotopic transplanted glioma models showed low uptake of ^18^F-FP-PEG_2_-β-Glu-RGD_2_ in normal brain tissue and high uptake in glioma tissue. The results demonstrate that ^18^F-FP-PEG_2_-β-Glu-RGD_2_ can be a potential tracer for PET imaging of cerebral and peripheral tumors.

## Conclusion

In this study, we successfully designed and synthesized a novel integrin α_v_β_3_ receptor-targeting symmetric homodimeric peptide, PEG_2_-β-Glu-RGD_2_, using a symmetric linker Fmoc-β-Glu(OSu)-OSu. ^18^F-FP-PEG_2_-β-Glu-RGD_2_ showed high tumor uptake and good tumor-to-background contrast in PC-3, A549 and C6 brain glioma tumor models. The symmetric homodimer tracer is easy to prepare and has high specificity in tumor models, which makes it a promising PET tracer for tumor angiogenesis imaging.
